# Expression pattern and prognostic potential of histamine receptors in epithelial ovarian cancer

**DOI:** 10.1007/s00432-022-04114-x

**Published:** 2022-06-25

**Authors:** Fabian B. T. Kraus, Nicole E. Topalov, E. Deuster, I. Hysenaj, D. Mayr, A. Chelariu-Raicu, S. Beyer, T. Kolben, A. Burges, S. Mahner, F. Trillsch, U. Jeschke, B. Czogalla

**Affiliations:** 1 Department of Gynecology and Obstetrics, University Hospital, LMU Munich, Munich, Germany; 2 Institute of Pathology, Faculty of Medicine, LMU Munich, Munich, Germany; 3 Department of Obstetrics and Gynecology, University Hospital Augsburg, Augsburg, Germany

**Keywords:** Ovarian cancer, Histamine receptors, Tumor microenvironment, Personalized medicine

## Abstract

**Purpose:**

Despite recent advances in the treatment of ovarian cancer (OC), long-term remissions remain scarce. For a targeted approach, prognostic markers are indispensable for predicting survival and treatment response. Given their association with multiple hallmarks of cancer, histamine receptors (HR) are emerging as promising candidates. Here, we investigate their expression pattern and prognostic value in OC.

**Methods:**

Specimens of 156 epithelial OC patients were collected during cytoreductive surgery at the Department of Obstetrics and Gynecology, LMU, between 1990 and 2002 and combined in a tissue microarray. Immunohistochemical staining of the HR H1, H2, H3 and H4 was quantified by an immunoreactive score and linked with clinico-pathological data by Spearman’s correlation. Via ROC curve analysis, optimal cut-off values for potential prognostic markers were defined. Overall survival (OS) was visualized in Kaplan–Maier curves and significances determined by log-rank testing. A Cox regression model was applied for multivariate analysis.

**Results:**

HR H3 and H4 expression was restricted to the cytosol of OC cells, while H1 was also present in the nucleus. A significant association between HR H1, H3 and H4 expression with several clinico-pathological parameters was revealed. In addition, HR H1 and H3 expression correlated positively, HR H4 expression negatively with OS. In addition, HR H3 was identified as independent prognostic marker for OS. HR H2 expression had no prognostic value.

**Conclusions:**

HR H1, H3 and H4 could serve as potential predictors for OS of OC patients. Further research is warranted to elucidate their pathophysiologic role and their predictive and therapeutic potential in OC.

**Supplementary Information:**

The online version contains supplementary material available at 10.1007/s00432-022-04114-x.

## Introduction

Ovarian cancer (OC) ranks first in lethality in gynecologic cancers and is the seventh cause of tumor-associated morbidity and mortality among women worldwide (Siegel et al. 2020; Momenimovahed et al. 2019). In the last 20 years, the prevalence and incidence of OC has significantly increased, the latter of which is expected to rise further by about 47% by 2040 (Sung et al. 2021). Due to the relatively late onset of symptoms and a lack of reliable screening methods, OC is often diagnosed in an advanced stage with a risk of relapse of approximately 85% in the first 10 years after diagnosis (Redondo et al. 2021; Czogalla et al. 2019). A dramatic decline in 5 year survival rates from 86% in *Fédération Internationale de Gynécologie et d’Obstétrique* (FIGO) I to 26% in FIGO IV patients parallels this diagnostic delay (Torre et al. 2018).

Apart from FIGO stage, further prognostic factors include histological subtype, tumor grade, patient’s age at diagnosis and, most importantly, the presence of residual disease following primary debulking surgery (Du Bois et al. 2009; Aletti et al. 2006; Dembo et al. 1990). For several decades, first-line therapy has consisted of cytoreductive surgery prior to an adjuvant platinum-based chemotherapeutic regimen (Mahner and Pfisterer 2013). More recently, targeted therapies comprising, e.g., the anti-vascular endothelial growth factor (VEGF) antibody Bevacizumab or poly-adenosine–diphosphate–ribose–polymerase (PARP) inhibitors were included as maintenance therapies for a subgroup of patients with at least a partial response to platinum-based chemotherapy (Moore et al. 2018; Trillsch et al. 2022). Yet, despite those significant therapeutic advances, 5 year survival rates remain poor with only a modest increase from about 45 to 50% within the past 20 years (Shabir and Gill 2020).

Histamine receptors (HR) increasingly attract attention as they modulate cell proliferation, cell invasion, apoptosis, tumor vascularization and immune response (Nguyen and Cho 2021), which have become known as hallmarks of cancer (Medina and Rivera 2010; Hanahan and Weinberg 2011).

Histamine is a multifunctional endogenous biogenic monoamine, synthesized from the essential amino acid histidine by the enzyme histidine decarboxylase (Nguyen and Cho 2021). It acts as a neurotransmitter in the nervous system or as a local mediator of inflammation. Four G-protein coupled receptor subtypes, the HR H1, H2, H3 and H4, mediate histamine effects through multiple pathways (Parsons and Ganellin 2006).

Depending on various factors, such as the exact tumor entity, histamine concentration, HR exposition, and target cell type, histamine can exert pro- as well as antitumorigenic effects (Blaya et al. 2010; Massari et al. 2020). In cholangiocarcinoma, for example, HR H3 signaling was shown to sustain tumor growth by d-myo-inositol 1,4,5-trisphosphate (IP_3_)/Ca^2+^/protein kinase C (PKC)-dependent ERK1/2 dephosphorylation (Francis et al. 2009). In non-small cell lung cancer, on the contrary, it exerts antitumoral effects via the PI3K/Akt/mTOR and MEK/ERK signaling pathway (Zhao et al. 2021).

The context-dependent ambiguity of histamine function is reflected by diverging pre-clinical and clinical observations. In a phase III, multicenter, randomized clinical trial on the addition of histamines to Interleukin-2 based therapies of advance stage melanoma patients, for example, Agarwala et al. 2002) observed an increase in response rates from 20 to 38% and in median survival from 154 to 283 days. In contrast, systemic histamine supplementation in colorectal cancer bearing mice was reported to promote tumor growth (Tomita and Okabe 2005). Consistently, trials on the preoperative treatment of colorectal cancer patients with the HR antagonist famotidine during the week before surgery yielded decreased recurrence rates and an augmented overall survival (OS). These reports further underline the diverging roles of histamines and antihistamines in different cancers (Kapoor et al. 2005).

The effects of HRs on the development and growth of different cancer types are widely acknowledged yet context-dependent. Specifically in the context of epithelian ovarian cancer (EOC), further understanding of their functional role is lacking. Therefore, our work investigates the expression patterns and prognostic relevance of all four currently known HRs in 142 EOC patients.

## Materials and methods

### Tissue microarray

Tumor specimens of 156 EOC patients were collected during cytoreductive surgery, carried out at the Department of Obstetrics and Gynecology, LMU, between 1990 and 2002. Following histopathological diagnosis confirmation, they were combined in a tissue microarray (TMA). All patients in this study underwent standard therapy for OC. In detail, this consisted of debulking surgery followed by adjuvant therapy. Only patients with pathologically confirmed EOC were included. Corresponding clinical data was gathered from the patients’ charts. Regular follow-up data was obtained by the Munich Cancer Registry.

Following tissue sampling, tumors were formalin-fixed and paraffin-embedded (FFPE). Representative tumor areas were biopsied for assembly in the TMA and their histopathological subtype was assessed by gynecological pathologists at the Department of Pathology at the LMU Munich, Germany. The TMA comprises 110 serous, 21 endometrioid, 13 mucinous and 12 clear cell ovarian cancer specimens, which were graded according to the currently valid World Health Organization classification criteria. Endometrioid tumors were graded from G1 to G3 and mucinous ovarian cancer samples, which currently still lack distinct WHO-approved classification criteria, were graded analogously. Serous ovarian cancer specimens were subdivided into high- and low-grade tumors and clear cell carcinomas automatically classified as G3.

Details on the distribution of selected clinico-pathological characteristics of our TMA-cohort can be found in Table 1.

**Table 1:** Clinico-pathological features of the 156 epithelial ovarian cancer patients included in our tissue microarray

Clinico-pathological parameters	*n*	Percentage (%)
Histology		
Serous	110	70.5
Clear cell	12	7.7
Endometrioid	21	13.5
Mucinous	13	8.3
Primary tumor expansion		
TX	1	0.6
T1	40	25.6
T2	18	11.5
T3	97	62.3
Nodal status		
pNX	61	39.1
pN0	43	27.6
pN1	52	33.3
Distant metastasis		
pMX	147	94.2
pM0	3	1.9
pM1	6	3.8
Grading serous		
low	24	21.8
high	80	72.7
Grading endometrioid		
G1	6	28.6
G2	5	23.8
G3	8	38.1
Grading mucinous		
G1	6	46.2
G2	6	46.2
G3	0	0
Grading clear cell		
G3	12	100
FIGO		
I	35	22.4
II	10	6.4
III	103	66.0
IV	3	1.9
Age		
≤ 60 years	83	53.2
> 60 years	73	46.8

### Immunohistochemistry

For immunohistochemical staining the FFPE tissue microarrays were dewaxed in xylol for 20 min and subsequently washed in 100% ethanol. To avoid unspecific binding of the staining antibodies, tissue sections were blocked in methanol containing 3% H_2_O_2_ for 20 min and then carefully rehydrated in serial dilutions of ethanol (100, 70 and 50%) prior to a final washing step in distilled water. Next, the tissue slides were autoclaved for 5 min in sodium citrate buffer (0.1 M citric acid in 0.1 M sodium citrate, pH = 6) and washed twice for 2 min, respectively, in distilled water and phosphate buffered saline (PBS). To avoid unspecific staining reactions, the tissue specimens were incubated in a blocking solution for 5 min at room temperature (RT) prior to a staining step at RT for 16 h with the following primary antibodies: anti-Histamine H1 R Immunoglobulin G (IgG) antibody (GeneTex, Irvine, USA), anti-HR H2 IgG antibody (Abcam, Cambridge, UK), anti-HR H3 IgG antibody (Origene, Rockville, USA) and anti-HR H4 IgG antibody (Origene, Rockville, USA). Following this, the samples were washed twice in PBS and a post-block reagent (Reagent 2, ZytoChem Plus horseradish peroxidase (HRP) Polymer System [mouse/rabbit], Zytomed, Berlin, Germany) was applied for 20 min at RT, followed by another washing cycle in PBS. The slides were, finally, incubated with an HRP-polymer conjugated anti-mouse/anti-rabbit antibody (Reagent 3, ZytoChem Plus HRP Polymer System [mouse/rabbit], Zytomed, Berlin, Germany). After another washing step in PBS, 3’3 diaminobenzidine (DAB) and the corresponding substrate buffer (Liquid DAB and Substrate Chromogen System, DAKO, Munich, Germany) were added to the tissue specimens. The staining reaction was halted by another washing step with distilled water. Mayer’s acidic haemalum (Waldeck, Münster, Germany) was applied for counterstaining. For dehydration, the tissue sections were washed with increasing concentrations of ethanol (first 70, then 96 and 100% ethanol) and, subsequently, with xylol. To ensure staining specificity and as an inherent quality control for the staining reaction, hepatic, colonic and placental tissue served as negative and positive controls, respectively (online resource). As a second method to monitor for unspecific antibody binding, additional tumor sections were stained with the corresponding isotype controls.

### Staining evaluation and statistical analysis

On the basis of the immunohistochemical staining, the immunoreactive (IR) score (Remmele and Stegner 1987) was applied for the semi-quantitative assessment of protein expression using a Leitz photomicroscope (Wetzlar, Germany). The percentage of stained cells (with 0: = unstained; 1:= $\le$ 10%; 2: = 11–50%; 3: = 51–80%; 4$:= \ge$ 81%) was multiplied with the predominant optical staining intensity (with 0: = no staining intensity; 1: = weak; 2: = moderate; 4: = strong). For each immunohistochemical staining a separate IR score was calculated, taking into account the different distribution patterns of the protein within each cell. Therefore, the score values differ with respect to the cellular compartment assessed, i.e., nucleus and cytoplasm. In order not only to represent the median staining intensity but also its variation within one tissue specimen as realistically as possible, we indicate the statistical data range in brackets in addition to the median IRS using the following format: median IR score (minimum score; maximum score).

Statistical analysis was carried out using International Business Machines Corporation (IBM) Statistical Package for Social Sciences (SPSS) Statistics 28.0 (IBM Corporation, Armonk, New York, USA). Bivariate correlations between protein expression and clinico-pathological patient data were calculated using Spearman’s analysis (Spearman 1987). For visualization of the OS, Kaplan–Meier curves were used. Log-rank testing was performed to check for statistical significance (Dinse and Lagakos 1982). Optimal cut-off values in survival analysis, stratified for the suggested prognostic markers, were estimated via a receiver operating characteristic (ROC) curve analysis, which is deemed a reliable and recognized method for cut-off value definition. By means of the Youden Index, cut-off values were additionally optimized to balance the sensitivity and specificity of the prognostic marker (Youden 1950; Fluss et al. 2005). A Cox regression model of the investigated parameters was employed for multivariate analyses (Cox 1972). Differences between experimental groups were considered statistically significant at a *p* value of $\le$ 0.05.

### Ethical approval

All tissue samples derive from material collected during cytoreductive surgery and stored in the archives of the Department of Gynecology and Obstetrics, Ludwig–Maximilians-University (LMU), Munich, Germany. Tumor tissue was only cleared for scientific use after the completion of a histopathological assessment and the full anonymization of patient data during all experimental and analytical stages. This study design, in accordance with the guidelines of the Ethics Committee of the LMU, made it possible to waive individual written consent as well as individually signed permission to publish. Ethical approval was granted under the numbers 227–09, 18–392 and 19–972. Moreover, all experiments were carried out in compliance with the standards of the Declaration of Helsinki in 1975.

## Results

### Histamine receptor expression pattern in epithelial ovarian cancer cells varies between different cellular compartments and correlates with clinico-pathological characteristics

**Fig. 1:**
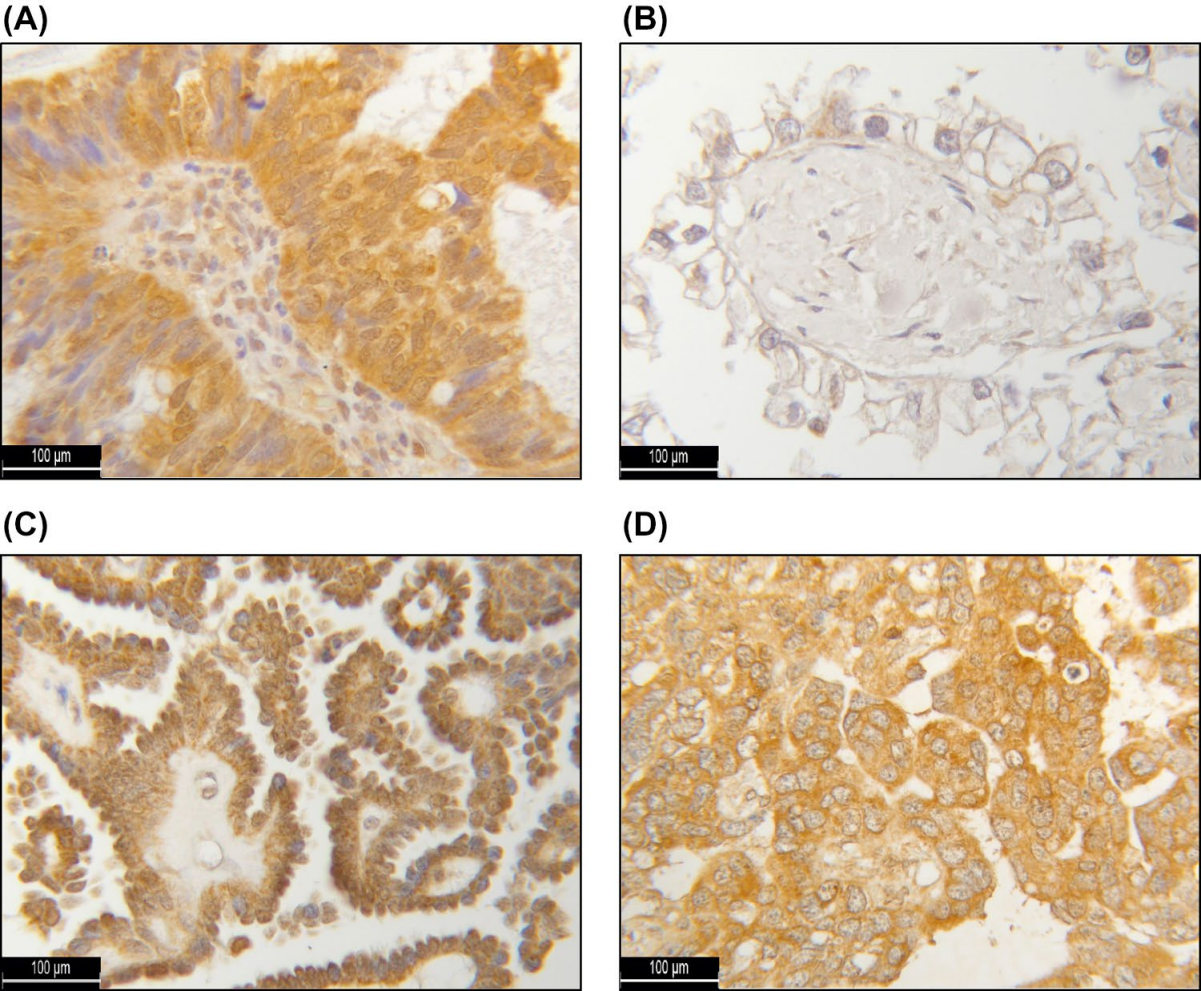
Immunohistochemical staining of the histamine receptors H1 (**A**), H2 (**B**), H3 (**C**) and H4 (**D**). As a positive (negative) control, hepatic (placental) tissue was used for the HR H1 staining, placental tissue for the HR H2 staining and colonic (placental) tissue for the HR H3 and HR H4 staining (online resource)

To investigate the prognostic and therapeutic value of the HR subtypes H1–H4 for EOC patients, immunohistochemical staining of all 156 tissue sections of our TMA was examined. HR expression intensity and distribution pattern could be assessed in 142 cases (91%), while 14 tissue samples had to be excluded due to poor tissue quality. The selected patient cohort had a median age of 58.8 ± 12.9 years, ranging from 20.7 to 88.0 years, while their median OS amounted to 60 ± 56.5 months. Immunohistochemistry revealed localization-dependent differences in intracellular HR H1 expression. For cytosolic HR H1, the median (range) IR score amounted to 5 (0; 12) and to 1 (0; 12) for nuclear HR H1. No relevant nuclear expression of the HR H2, H3 and H4 could be detected. The median (range) IR scores for cytosolic HR H2, H3 and H4 reached 5 (2; 12), 7 (0; 12) and 9 (4; 12), respectively (Fig. 1).

Cut-off values for marker positivity were determined for each HR via ROC-curve analysis and defined as follows (Table 2).

**Table 2:** Cut-off values for immunohistochemical histamine receptor positivity

Receptor subtype	Cut-off value for positivity	Log-rank significance	*n*
HR H1 (cytosolic)	> 6	0.007	18
HR H1 (nuclear)	> 1	0.016	29
HR H2	–	No significance	
HR H3	> 8	0.017	21
HR H4	> 6	0.047	99

For HR H2 no cut-off value was deemed significant based on Log-rank analysis. A significant correlation of nuclear with cytosolic HR H1 positivity (*p* = 0.026; *Cc* = 0.206), just as of cytosolic HR H1 with HR H3 positivity (*p* = 0.001; *Cc* = 0.310) was detected. No correlation could be found of HR H2 or H4 expression with any other HR subtype.

In addition, a correlation analysis of HR expression and clinico-pathological features revealed a significant inverse correlation between a positive nuclear HR H1 expression (IRS > 1) and high-grade histology, pathological tumor staging, patient age at diagnosis and FIGO stage. A positive nuclear HR H1 expression, in contrast, directly correlated with a mucinous histopathological subtype. Moreover, a positive cytosolic HR H1 expression (IRS > 6) is significantly correlated with a young age at diagnosis and low-grade histology. Furthermore, a significant positive correlation could be detected between HR H3 expression (IRS > 8) and endometrioid or low-grade serous subtype. Positive cytoplasmatic HR H4 staining (IRS > 6) was positively correlated with a high-grade serous subtype and FIGO stage (Table 3).

**Table 3:** Correlation of histamine receptor positivity with clinicopathological parameters

Receptor subtype	Clinicopathological feature	*p* value	Correlation coefficient
Nuclear HR H1	High-grade histology	0.003	− 0.263
	Patient age at diagnosis	0.033	− 0.188
	FIGO stage	0.009	− 0.231
	Pathological tumor stage	0.001	− 0.289
	Mucinous subtype	0.001	0.291
Cytosolic HR H1	Age at diagnosis	0.032	− 0.193
	Histopathological tumor grade	0.031	− 0.194
Cytosolic HR H3	Endometrioid subtype	0.030	0.195
	Low-grade histology	0.006	0.248
Cytosolic HR H4	High-grade histology	0.001	0.316
	FIGO stage	0.029	0.208

### Overall survival of epithelial ovarian cancer patients correlates positively with nuclear HR H1 and cytosolic HR H1 and HR H3 staining and inversely with cytosolic HR H4 expression.

**Fig. 2:**
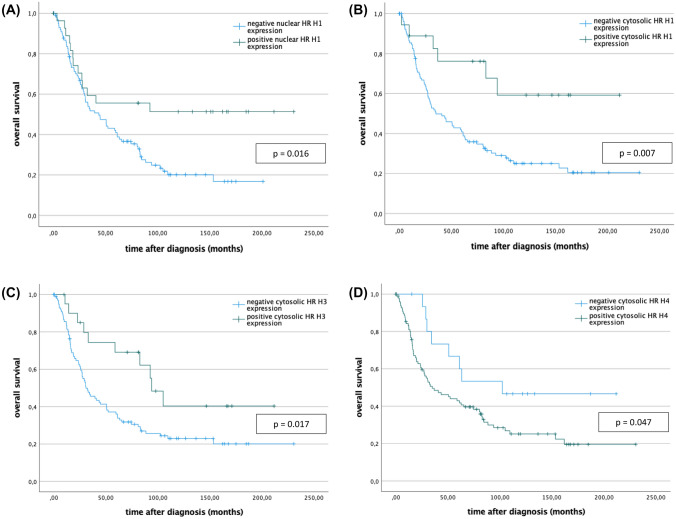
Positive nuclear (**A**) and cytosolic (**B**) HR H1 staining as well as positive cytosolic HR H3 (**C**) staining positively correlate with an increased overall survival. HR H4 positivity, in contrast, is inversely correlated with overall survival (**D**). Histamine receptor positivity and negativity was defined according to the cutoffs established in Table 2. For statistical analysis a log-rank test was performed. Censoring events were marked in the graphs ( +)

To further delineate the prognostic relevance of the HR-expression for the OS of EOC patients, univariate analysis was performed. For a positive nuclear HR H1 staining (median OS = 131.4 months vs. 70.6 months in nuclear HR H1 negative tumors; *p* = 0.016; Fig. 2A), as well as for a positive cytosolic HR H1 (median OS = 145.5 months vs. 80.6 months; *p* = 0.007**;** Fig. 2B), and for a positive HR H3 staining (median OS = 121.2 vs. 75.6 months; *p* = 0.017; Fig. 2C) we found a significantly prolonged OS as compared to EOC patients with HR scores below the cutoffs. For HR H4 positivity (IRS > 6), we found a significantly reduced median OS of only 80.3 months vs. 125.2 months in the HR H4 negative cohort (*p* = 0.047**;** Fig. 2D).

### Positive cytosolic HR H3 expression and clinico-pathological parameters are independent prognostic factors for overall survival

**Fig. 3:**
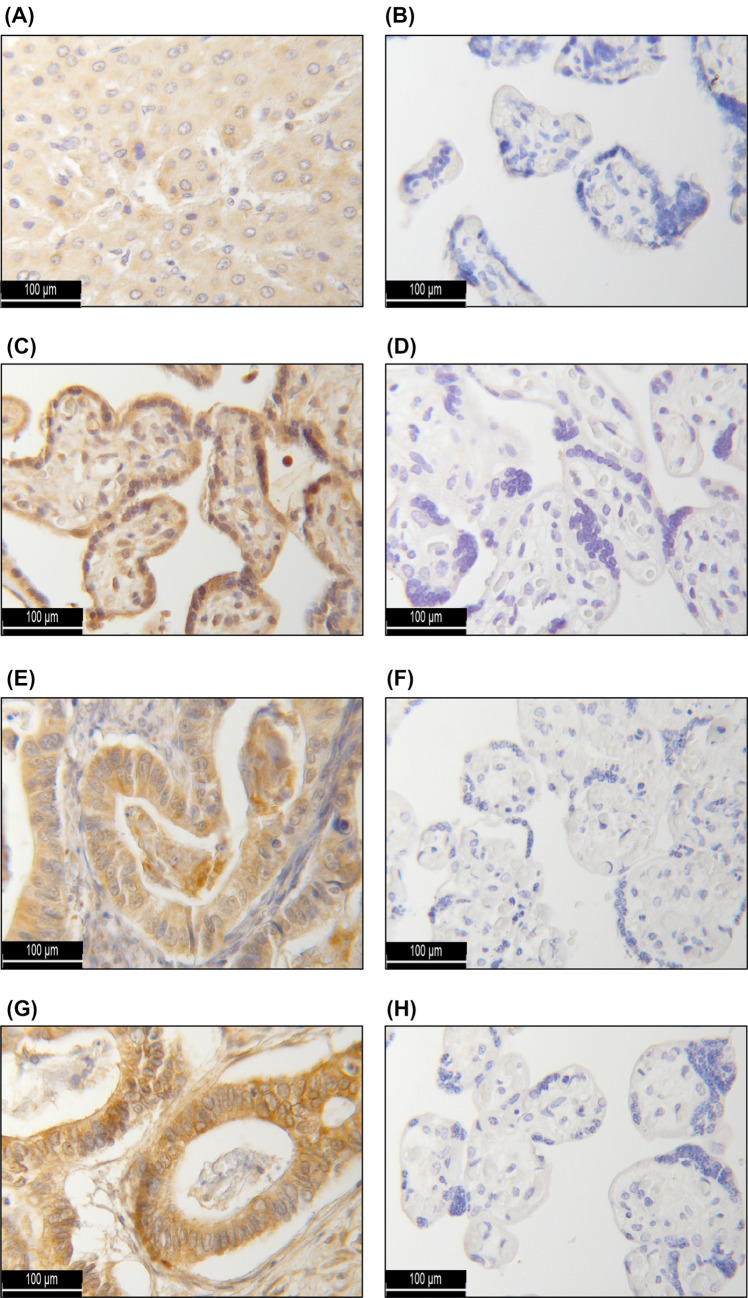
Immunohistochemical staining controls: As a positive (negative) control for immunohistochemical analysis, hepatic (**A**) (placental (**B**)) tissue was used for the HR H1 staining, placental tissue as both, the positive (**C**) and negative (**D**) control for the HR H2 staining and colonic (**E**) (placental (**F**)) tissue for the HR H3 staining. For the HR H4 staining, too, colonic (**G**) and placental (**H**) tissue were employed.

For the identification of independent factors prognostic for OS, a multivariate Cox regression analysis was performed (Table 4). Thereby, FIGO state (I, II vs. III, IV; *p* < 0.001) and histological grading (1 and 2 vs. 3; *p* = 0.023) were confirmed as independent prognostic factors. In addition, cytosolic HR H3 expression (IRS > 8) could be identified as a novel and statistically independent marker for a reduced OS (*p* = 0,005). In contrast, this analysis did not yield significant results with regards to the patients’ age at diagnosis, nuclear HR H1 expression and the cytosolic HR H1 and H4 expression (Fig. 3).

**Table 4:** Multivariate Cox regression analysis of all ovarian cancer patients with an assessable HR status (*n* = 142) and their clinico-pathological characteristics

Covariate	Hazard Ratio	95% CI	*P* value
FIGO (I, II vs. III, IV)	3.921	1.711–8.983	0.001**
Grading (1 and 2 vs. 3)	2.510	1.244–5.065	0.010*
Patients’ age (continuous)	1.016	0.991–1.041	0.214
Nuclear HR H1 expression	1.009	0.871–1.169	0.908
Cytosolic HR H1 expression	1.026	0.892–1.181	0.718
Cytosolic HR H3 expression	0.865	0.783–0.956	0.005*
Cytosolic HR H4 expression	1.086	0.977–1.207	0.125

Nuclear and cytosolic HR expression were each quantified by the means of the immunoreactive score. Cut-off values for marker positivity were defined as indicated in Table 2

*CI* confidence interval

Significant independent factors for overall survival are highlighted with asterisks (*p < 0.05; **p ≤ 0.001).

## Discussion

Despite its unequivocal influence on tumor cell proliferation, de-differentiation and immune surveillance, the exact role of histamine and its receptors remains ambiguous. In multiple *in* *vitro* studies and clinical trials, histamine was shown to exert either a pro- or an anti-tumorigenic effect. Influencing factors include the exact histamine dose, the respective HR involved, its consecutive signaling pathways, as well as the individual tumor or target cell type (Perz and Ho 2008; Kapoor et al. 2005). Given their controversial roles, further studies will be required to assess the actual diagnostic, prognostic and therapeutic value of HR for oncologic patients. Interestingly, literature on the relevance of HR for EOC patients remains particularly scarce. In 1995, Chanda and Ganguly showed that histamine concentrations were significantly elevated in human ovarian, endometrial and cervical carcinoma compared to their adjacent tissues (Chanda and Ganguly, 1995). In a large population-based study, however, Lacey et al. (2004) postulated that the regular use of HR-antagonists for more than 5 years increases the lifetime risk for ovarian cancer.

Due to the context-dependent ambiguity of earlier HR-data and a lack of further EOC-specific literature, the clinical relevance of the abovementioned findings and especially the prognostic significance of the HR H1 to H4 expression remain largely inconclusive.

In our present analysis we report a positive correlation between high HR H1 expression (IRS > 6 for cytosolic and IRS > 1 for nuclear HR H1 positivity) and prognostic relevant clinico-pathological features, such as FIGO stage, age at diagnosis and recurrence free or overall survival. In addition, the significant correlation of HR H1 expression with mucinous and high-grade serous histology shown in our experiments might serve as a tool to enhance the diagnostic certainty for the histological distinction between high- and low-grade serous subgroups.

In contrast to the newly-established link between HR H1 expression and pathological subtype described here, a functional link between histological grading and HR H1 signaling has already been postulated and is said to trigger tumor growth differently in low- and high-grade OC cells (Batra and Fadeel 1994). In 1994, Batra and Fadeel reported an HR H1-activation dependent rise of intracellular calcium concentration with a subsequent increase in the proliferation of SKOV-3 ovarian cancer cells. Analogous experiments were repeated in the more differentiated OVCAR-3 cell line. In contrast to the SKOV-3 model, calcium ions in OVCAR-3 cells are not exclusively freed from intracellular storage pools, but also derive from the extracellular space through a transmembranous influx into the cancer cells. Additional in vitro experiments will be necessary to further differentiate the exact signaling pathways leading to cell proliferation in response to the activation of certain HR subtypes.

Moreover, histamine receptors, such as HR H1, are not only exposed on the cell surface and expressed in the cytosol but can also be detected in the nucleus. This nuclear HR H1 expression was, much like a positive staining for cytosolic HR H1, significantly associated with an augmented OS. Although the nuclear localization of HRs to date has been poorly investigated, a strong association between HR density on the cell surface and its ability to trigger and regulate certain signaling pathways could be established (Medina and Rivera 2010; Mitsuhashi et al. 1989; Fitzsimons et al. 2002). This might at least partly explain the sometimes-opposing effects of histamines on cancer cell proliferation, depending on the amount of available HR on the cell surface and their consecutive signaling potency. Analogously, in vitro experiments with hepatocellular carcinoma cells showed that, depending on the exact histamine dosage and its subsequent signaling strength, histamine exerts either a pro- or an anti-proliferative effect on tumor cells (Lampiasi et al. 2007).

In consistence with the strong association of cytosolic HR H1 with HR H3 expression, not only HR H1 but also HR H3 positivity (IRS > 8) is significantly correlated with an increased OS. Interestingly, HR H3 has mostly been investigated in the context of certain neurological conditions, such as Alzheimer’s or Parkinson’s, cerebral ischemia or sleep/wake disorders (Nieto-Alamilla et al. 2016; Tiligada et al. 2009; Hu and Chen 2012). Yet, although its signaling cascades, including cAMP formation, calcium accumulation and MAPK pathway stimulation, might be of ubiquitous importance, only little is known about the role of HR H3 in OC (Dimitriadou et al. 1994).

It is of note that, while HR H1 and HR H3 expression mostly seems to be positively correlated with the patients’ survival, a significant negative correlation between HR H4 (IRS > 6) expression and patient survival prevailed in our studies. This finding corresponds with the paradoxical effects of histamines even within one tumor entity. It could at least partly be explained by intercellular differences in the signaling pathways employed by each HR with regard to the wide spectrum of different cell types within the tumor microenvironment.

The tumor microenvironment consists of a complex combination of extracellular matrix and non-transformed cells. The latter include immune cells, stromal cells and endothelial cells, which do not only interact with each other, but also with the tumor cells themselves and vice versa (Nguyen and Cho 2021). Therefore, understanding the impact of the histamine receptor signaling on non-tumorous cells within the tumor microenvironment might also lead to indirect but nevertheless effective anti-cancer strategies (Hirata and Sahai 2017; Quail and Joyce 2013; Lee et al. 2020). In this context, the negative prognostic value of high HR H4 expression as opposed to the positive correlation between high HR H1 and HR H3 expressions and an increased OS might be explicable insofar as HR H4 plays a key role in mast cell chemotaxis and degranulation (Hofstra et al. 2003). Mast cells, in turn, have been reported to exert both a pro- and antitumoral function, depending on the individual tumor type and exact experimental or clinical conditions. For high-grade serous OC patients, Hodeib et al. (2016) demonstrated a strong association between the amount of activated stromal mast cells and a poor OS. This is paralleled by a particularly immunoreactive tumor microenvironment, characterized by an augmented regulatory T cell (T_reg_) infiltrate and M2 polarized macrophages, which might explain the low response rates to immunotherapeutic treatment approaches in these patients.

In summary, our work confirms the expression of the HR subtypes H1, H2, H3 and H4 in EOC. We could reveal a significant prognostic advantage for patients with HR H1 or H3 positive tumors, as a strong cytosolic and nuclear HR H1 expression as well as a high cytosolic HR H3 expression are associated with a significant survival benefit. In contrast, HR H4 positivity was correlated with decreased OS in our analysis.

However, these findings are, at least to a certain extent, subject to methodological limitations. Our TMA comprises specimens from 156 patients who were diagnosed between 1990 and 2002, thus potentially hindering the analysis of effects induced by recently implemented therapeutic changes. Nevertheless, our cohort, therefore, benefits from a long period for follow-ups. In addition, statistically significant effects observed in our first-line treatment cohort might eventually prove to be fundamental and robust and thus worth further investigation.

Additional studies will be needed to delineate the characteristics of different HR pathways further and might be the base for new OC therapies that rely on a specific modulation of HR signaling. Several HR-antagonists and -agonists have already been approved for clinical use, thus potentially narrowing the gap between bench and bedside. Given that radio sensitizing (Soule et al. 2010) and/or hormone modulating effects (Rossing et al. 2000) of antihistamines have already been established in the past, further in vitro and in vivo experiments are warranted not only to investigate the therapeutic potency of HR modulation, but also to unveil potentially synergistic effects of histamine receptor modulators with other treatment modalities.

## Supplementary Information

Below is the link to the electronic supplementary material.

Supplementary file1 (DOCX 21820 KB)
