# Supplementary material for: Expression pattern and prognostic potential of histamine receptors in epithelial ovarian cancer
Source: J Cancer Res Clin Oncol. 2022 Jun 25;149(6):2501–11. doi: 10.1007/s00432-022-04114-x (PMC10129941; doi:10.1007/s00432-022-04114-x)

**Online resource: Expression Pattern and Prognostic Potential of the Histamine Receptors in Epithelial Ovarian Cancer**

***Kraus FBT^1^, Topalov NE^1^, Deuster E^1^, Hysenaj I^1^, Mayr D^2^, Chelariu-Raicu A^1^, Beyer S^1^, Kolben T^1^, Burges A^1^, Mahner S^1^, Trillsch F^1^, Jeschke U^1,3^, Czogalla B^1^**

*^1^ Department of Gynecology and Obstetrics, University Hospital, LMU Munich, Munich, Germany.*

*^2^ Institute of Pathology, Faculty of Medicine, LMU Munich, Munich, Germany.*

*^3^ Department of Obstetrics and Gynecology, University Hospital Augsburg, Augsburg, Germany.*

**corresponding author: Fabian Kraus E-Mail: Fabian.Kraus@med.uni-muenchen.de*

**A) B)**


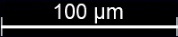

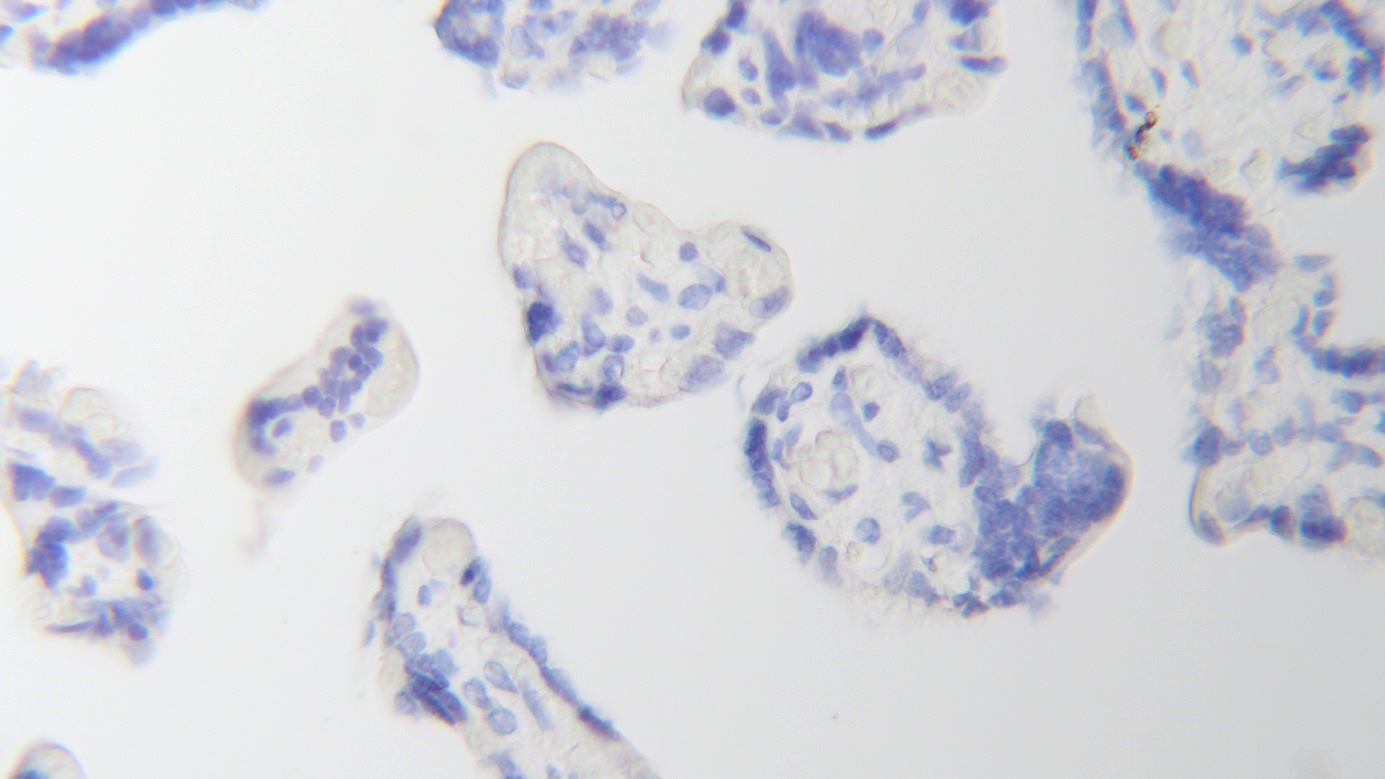


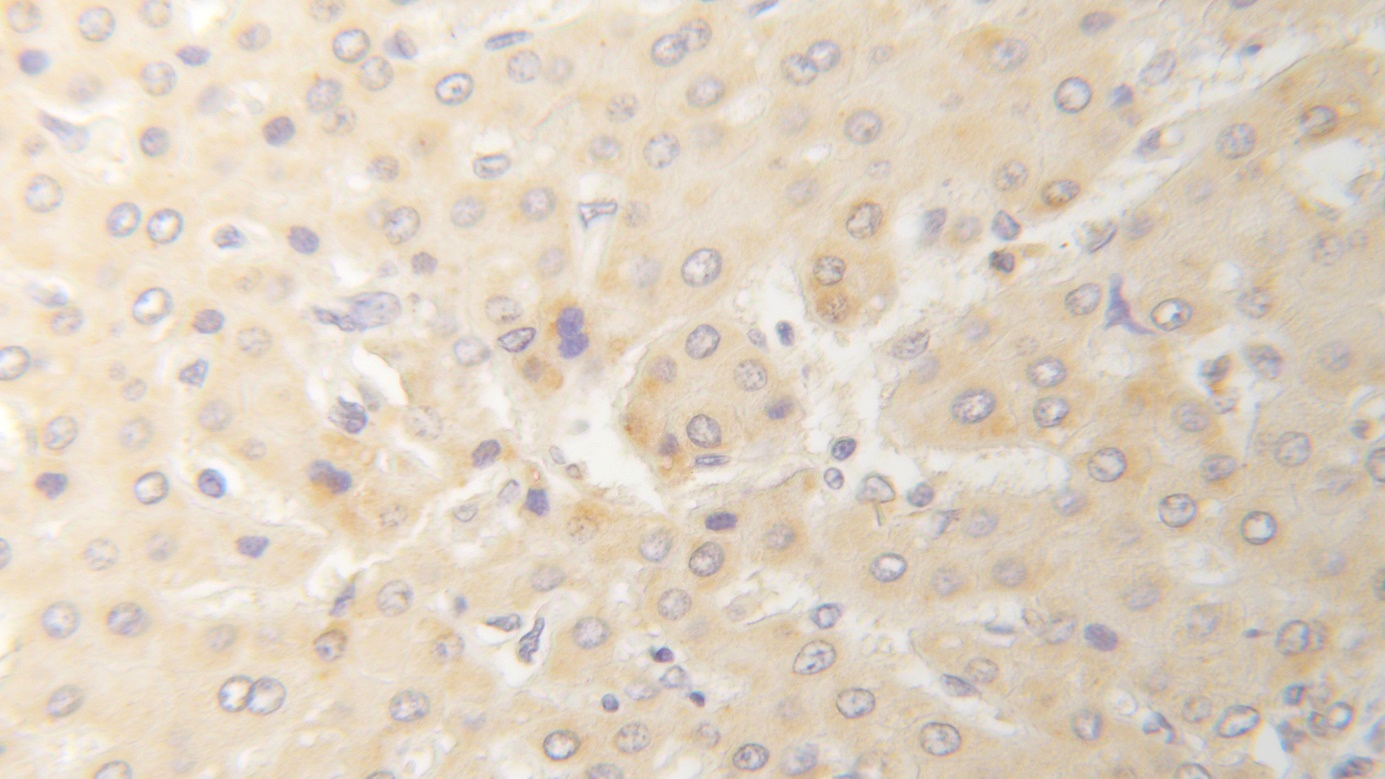

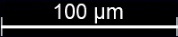


**C) D)**


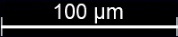

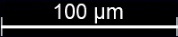

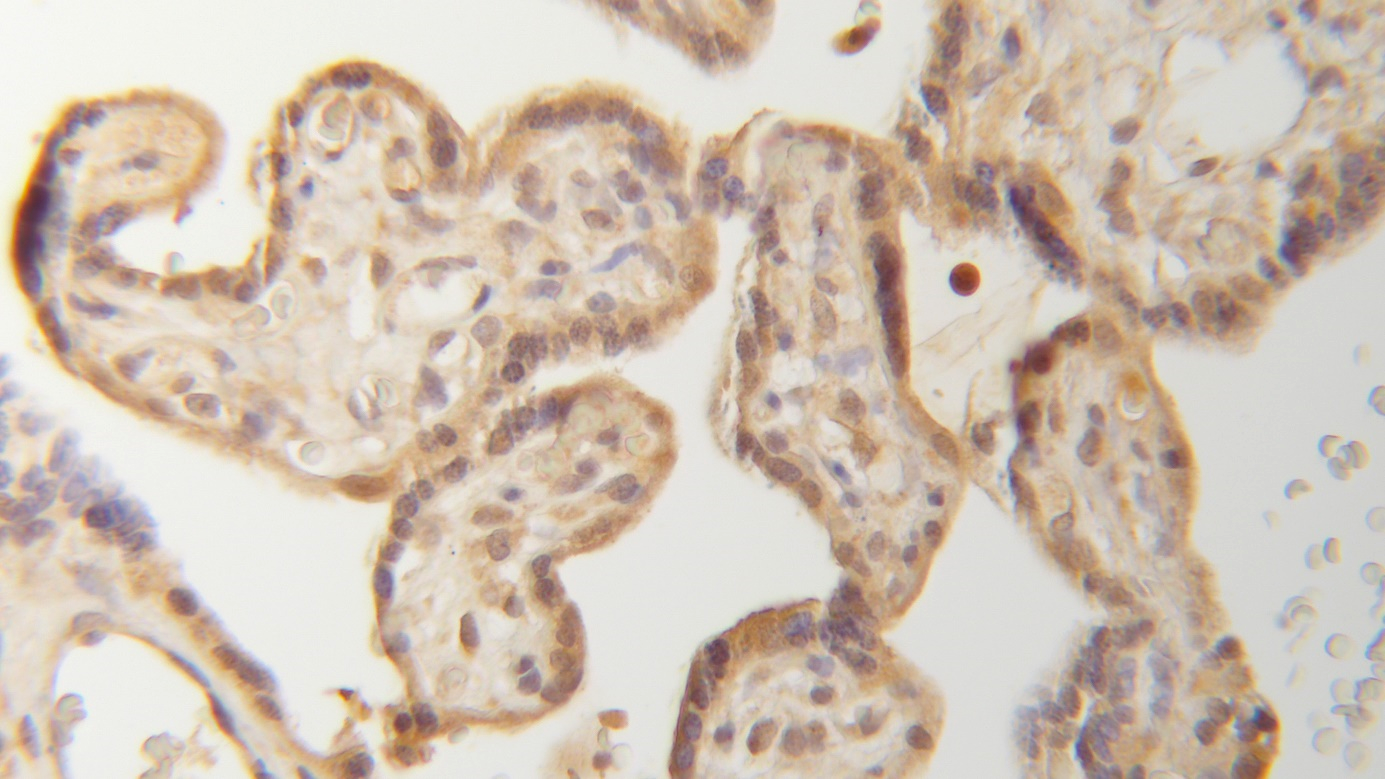

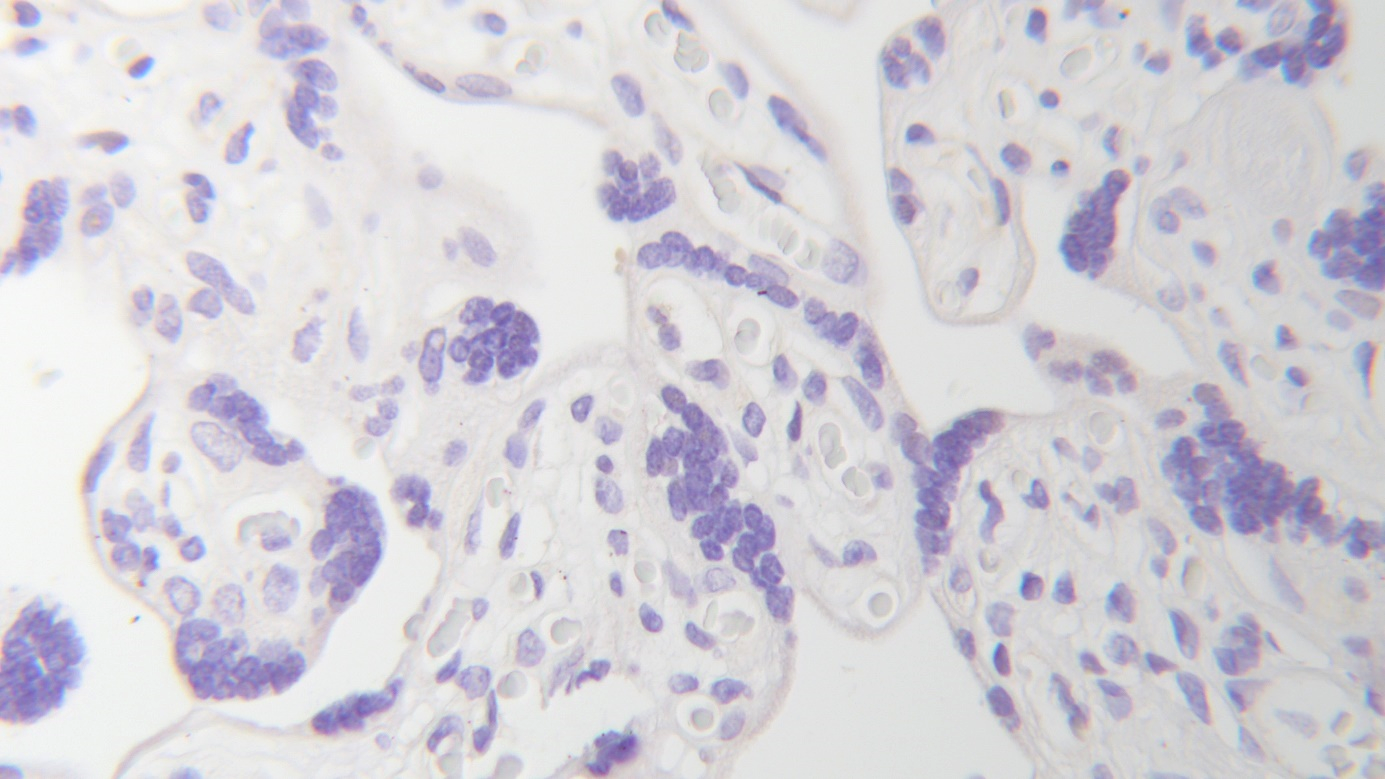


**E) F)**


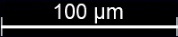

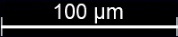

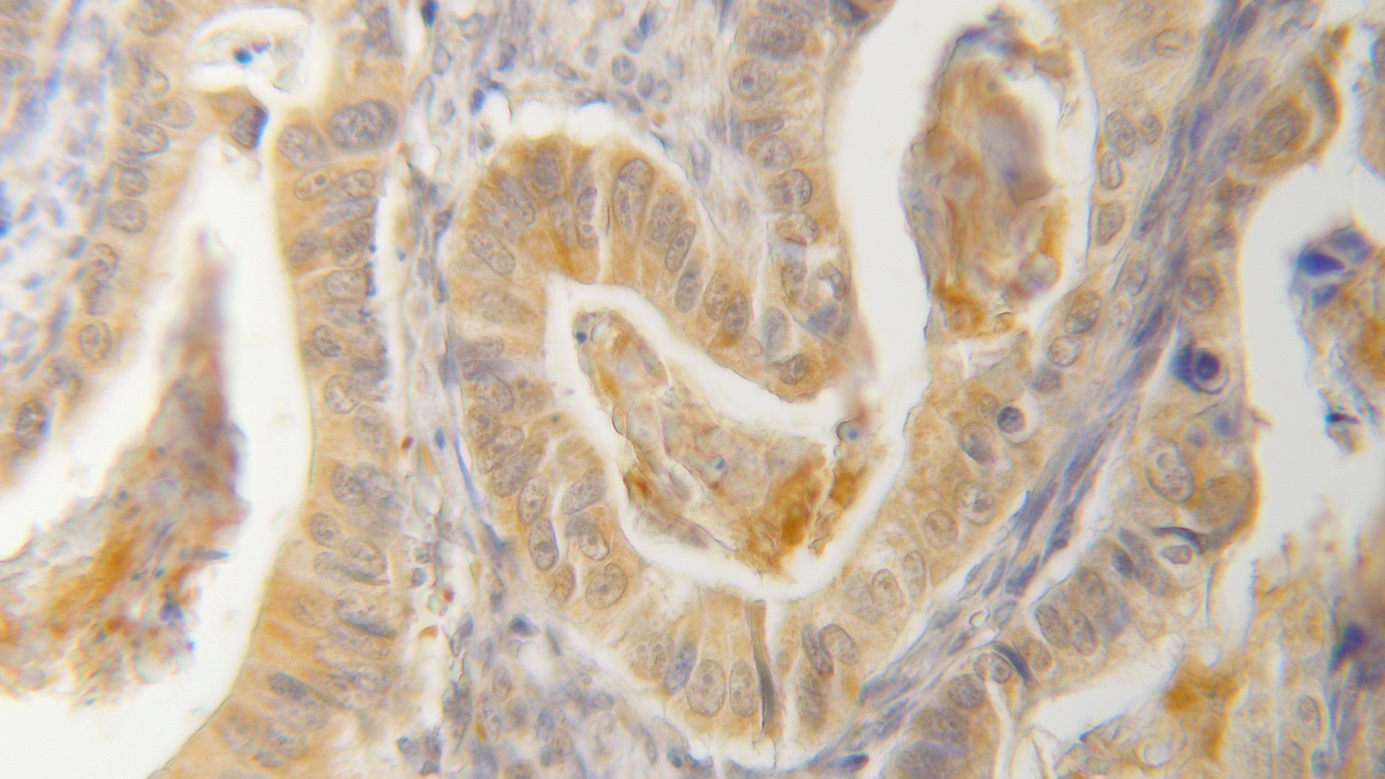

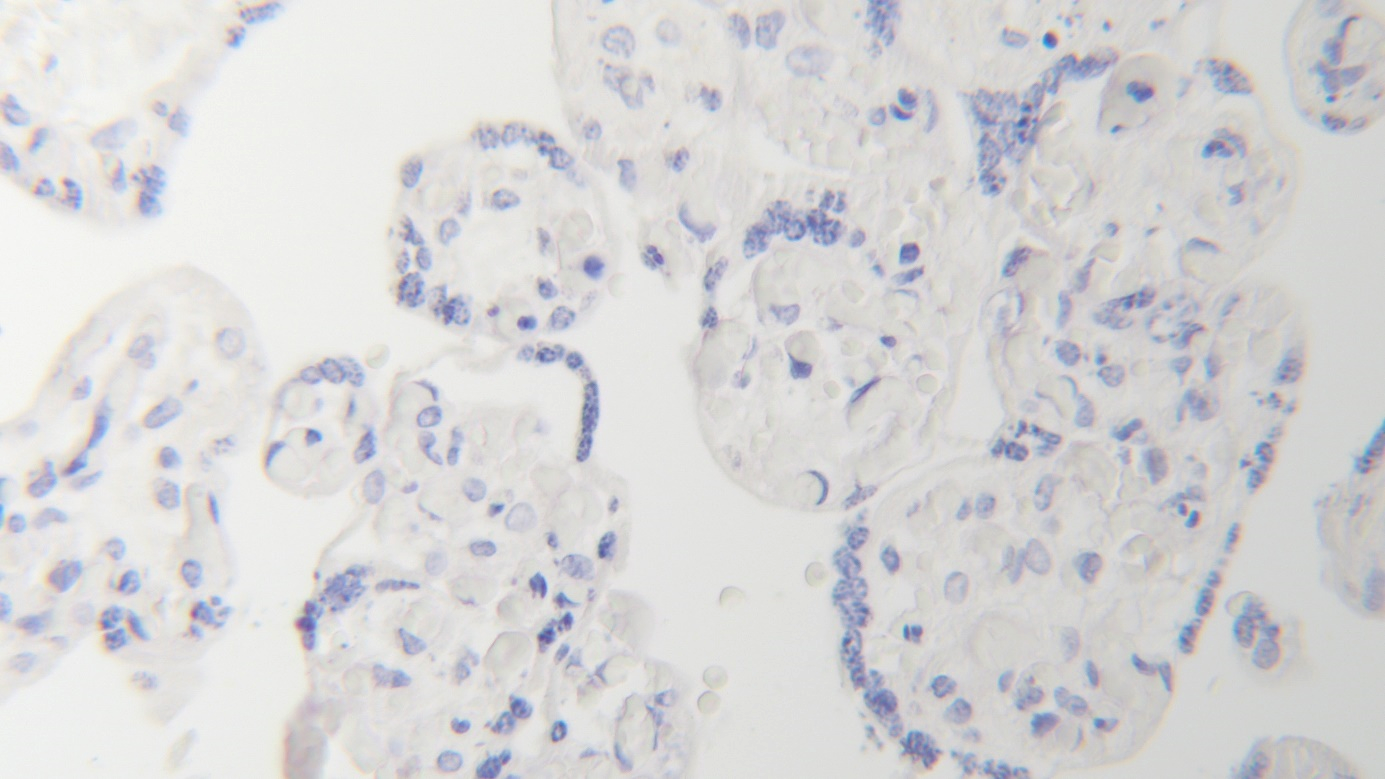


**G) H)**


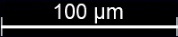

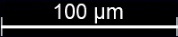

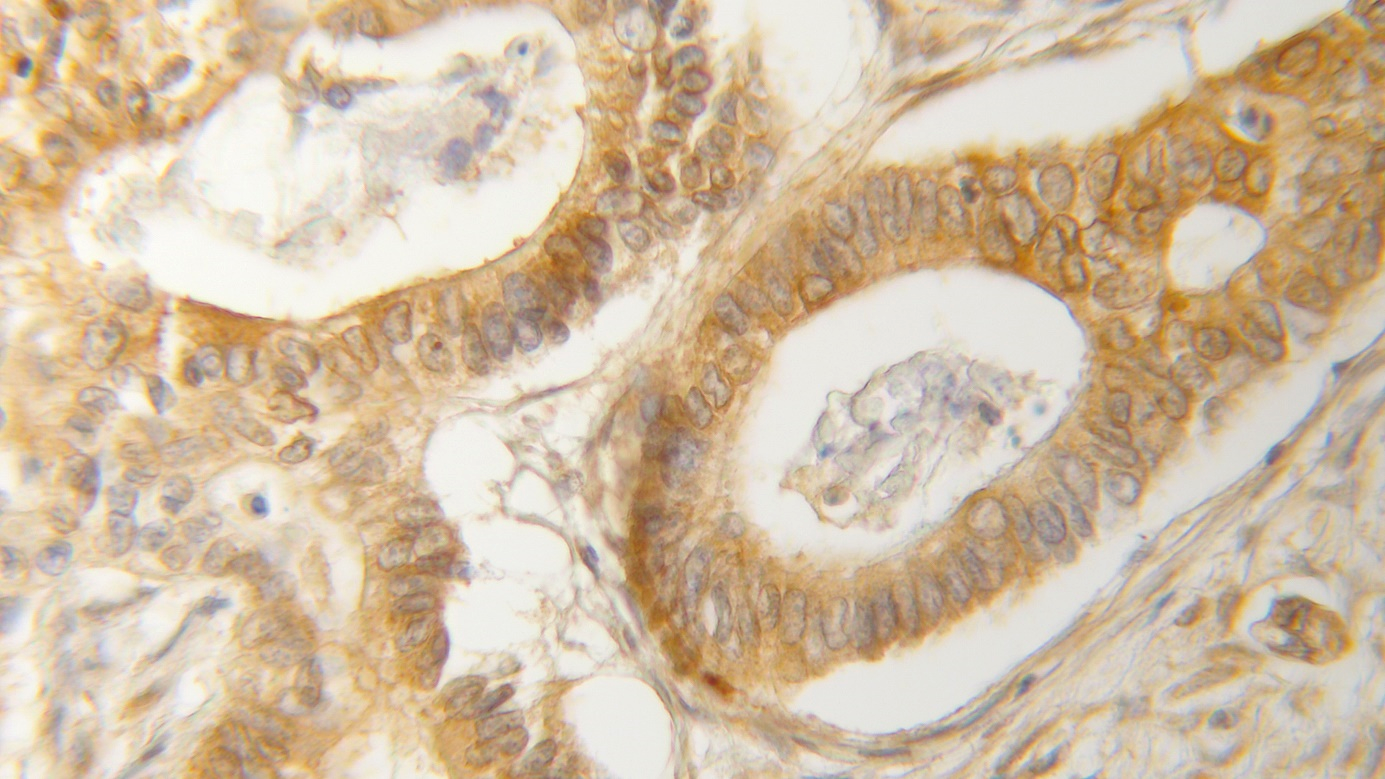

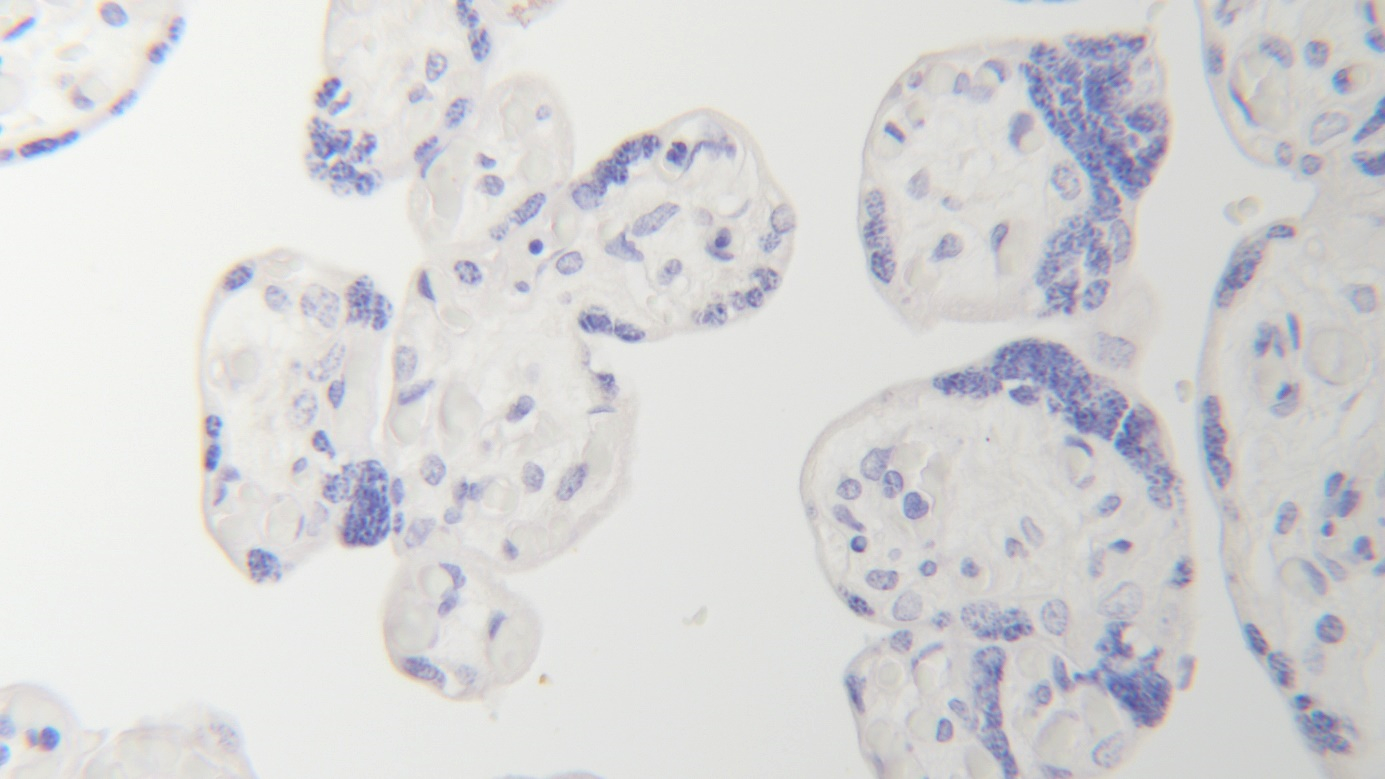

Supplement: Supplementary file 1 — Supplementary file1 (DOCX 21820 KB) [file 432_2022_4114_MOESM1_ESM.docx]
